# Trends of obesity and abdominal obesity in Tehranian adults: a cohort study

**DOI:** 10.1186/1471-2458-9-426

**Published:** 2009-11-23

**Authors:** Farhad Hosseinpanah, Maryam Barzin, Parvin Sarbakhsh Eskandary, Parvin Mirmiran, Fereidoun Azizi

**Affiliations:** 1 Obesity Research Center, Research Institute for Endocrine Sciences, Shahid Beheshti University of Medical Sciences, Tehran, Iran; 2 Research Institute for Endocrine Sciences, Shahid Beheshti University of Medical Sciences, Tehran, Iran; 3 Endocrine Research Center, Research Institute for Endocrine Sciences, Shahid Beheshti University of Medical Sciences, Tehran, Iran

## Abstract

**Background:**

Considering the increasing trend of obesity reported in current data, this study was conducted to examine trends of obesity and abdominal obesity among Tehranian adults during a median follow-up of 6.6 years.

**Methods:**

Height and weight of 4402 adults, aged 20 years and over, participants of the Tehran Lipid and Glucose Study (TLGS), were measured in 1999-2001(phase I) and again in 2002-2005(phase II) and 2006-2008 (phase III). Criteria used for obesity and abdominal obesity defined body mass index (BMI) ≥ 30 and waist circumference ≥ 94/80 cm for men/women respectively. Subjects were divided into10-year groups and the prevalence of obesity was compared across sex and age groups.

**Results:**

The prevalence of obesity was 15.8, 18.6 and 21% in men and 31.5, 37.7 and 38.6% in women in phases I, II and III respectively (p < 0.001). The prevalence of abdominal obesity in men was 36.5, 57.2 and 63.3% and in women was 76.7, 83.8 and 83.6% in the three periods mentioned (p < 0.001). Men aged between 20-29 years had highest increase rates of obesity and abdominal obesity in phase III in comparison with phase I (with a respective rates of 2.2- and 3.3-fold). In both sexes, an increased trend was observed between phases I and II, whereas between phases II and III, this trend was observed in men, but not in women.

**Conclusion:**

This study demonstrates alarming rises in the prevalences of both obesity and abdominal obesity in both sexes especially in young men, calling for urgent action to educate people in lifestyle modifications.

## Background

Obesity, general and abdominal, poses one of the greatest public health challenges for the 21st century with particularly alarming trends in several parts of the world [1]. Unhealthy diets and physical inactivity are the main contributors to overweight and obesity, which are among the leading risk factors for the major non-communicable diseases. The most significant consequences for health of overweight and obesity include hypertension and hyperlipidaemia, coronary heart diseases, ischemic stroke, type 2 diabetes, certain kinds of cancer [2]. In 2005, the estimated total numbers of overweight and obese adults worldwide, were 937 million and 396 million respectively [1], numbers that have doubled in comparison to 20 years ago [3]; by 2030, these figures are projected to be 1.3 billion and 573 million for overweight and obese adults respectively [1]. Recently there is a greater emphasis on abdominal obesity, as compared with general obesity, in relation to the risk of incidence of non-communicable weigh-related diseases [4]. Studies show the prevalence of abdominal obesity to be increasing along with general obesity [5]; for instance, in NHANES, during 10 years (from 1994 to 2004), an increase of 10% was observed in the prevalence of abdominal obesity among American adults [6].

In Iran, the prevalence of overweight and obesity in 2005 was reported to be 42.8% in men and 57% in women [7]; for 2015, the figures are predicted to be 54 and 74% respectively [8]. Studies have shown that the prevalence of abdominal obesity to range between 9.7 - 12.9% and 54.5 - 63.7% in Iranian men and women respectively [7,9]. There is however limited data available on this trend from Iran.

The Tehran Lipid and Glucose Study (TLGS) with median follow-up of 3.6 years [10] reported an increasing trend for prevalence of obesity and abdominal obesity in both sexes. We conducted this survey with the objective of determining this trend in Tehranian adults between the years 1999 and 2008 (with median follow-up of 6.6 years).

## Methods

### General information

The Tehran Lipid and Glucose Study was conducted to determine the risk factors of atherosclerosis among Tehran's urban population and to develop population-based measures to prevent the rising trend of diabetes mellitus and dyslipidemia. The design of this study encompasses three major components; phase I, a cross-sectional prevalence study of cardiovascular disease and associated risk factors and phases II and III, prospective follow-ups study for 20 years [11]. A multistage stratified cluster random sampling technique was used to select 15,005 people, aged 3 years or older, from district 13 of Tehran, the capital of the Iran; the district is located in the center of Tehran and the age distribution of its population is representative of the overall population of Tehran. From this population, only individuals above 20 years old, who participated in phase I in 1999-2001, phase II in 2002-2005 and phase III in 2006-2008 were chose. The interval between two assessments was at approximately 3.6-year intervals in this survey. The details of this study have been published elsewhere [12].

### Data collection

After excluding subjects aged < 20 years (n = 4637), those with missed values of weight, height, or other variables (n = 485), or lost to follow-up (n = 5481), data of 4402 subjects including 41.6% men (1835 persons) with a complete 6.6-year follow-up was used in this cohort study. There were 708 subjects in the 20-29 years age group, 1088 aged 30-39 years, 983 aged 40-49 years, 828 aged 50-59 years, 655 between 60 and 69 years, and 140 aged 70 years and over. In comparison to those who completed the follow-up, individuals lost to follow-up had lower value of systolic blood pressure (119 vs. 117 mmHg), fasting plasma glucose (4.99 vs. 4.94 mmol/L), age (44.5 vs. 41.5 years), BMI (27.1 vs. 26.5 Kg/m2) and waist circumference (89.1 vs. 87.2 cm) (p < 0.001).

This study was approved by the Research Ethics Committee of the Research Institute for Endocrine Sciences, Shahid Beheshti University of Medical Sciences, and informed written consent was obtained from all subjects and was conducted in accordance with the principles of the Declaration of Helsinki.

### Anthropometric measures

Weight and height were determined using a digital electronic weighing scale (Seca 707; range 0.1-150 kg, Hanover, MD) with an accuracy of up to 100 gr (the machine was regularly checked for precision after every 10 measurements) and tape meter stadiometer respectively. Waist and hip circumferences were measured using standard protocols by a trained individual. Waist circumference was measured at the level of the umbilicus and hip circumference was measured over light clothing at the widest girth of the hip. Body mass index [BMI = weight (kg)/height2 (m2)] and waist-to-hip ratio [WHR = waist circumference (cm)/hip circumference (cm)] were calculated; according to the International Obesity Task Force (IOTF) guidelines, overweight was determined as BMI > 25 and < 30, obesity was defined as BMI ≥ 30 [13], WC ≥ 94 cm in men and ≥ 80 cm in women was considered as the cut-offs for determining abdominal obesity [14].

### Statistical analysis

All variables are expressed as mean (SE) or percent. The means between 3 phases were compared using the repeated measurement test and comparison of overweight, obesity and abdominal obesity percentage between 3 phases was done using the Cochran test. Bonferroni correction was used for multiply comparison between these phases. In each phase, logistic models were developed to evaluate the role of sex in prediction of overweight, obesity and abdominal obesity separately. Age adjusted prevalence was estimated with the reference population group of Tehran according to the data from the 2006 census. All analyses were stratified by sex. All tests for statistical significance were two-tailed and performed assuming a type I error probability of < 0.05. All data were analyzed by the SPSS soft ware package (SPSS for Windows; SPSS Inc., Chicago, IL, USA; Version 16.00).

## Results

The mean ages of subjects in the 3 phases were 44.5, 48 and 50.9 years respectively. Of 4402 study participants, of 58.4% (n = 2567) were women. Mean Body Mass Index (BMI) was 27.1, 27.9 and 28.2 Kg/m2 in phase I, II and III, respectively; and mean WC was 89.1, 93.3 and 93.9 cm, respectively.

Mean (SE) for BMI, WC and WHR increased significantly in all age groups, in both sexes, between phase I and III. Whereas in men, WC increased in all age groups between the mentioned phases, increases for women were observed only between phases I and II (Table 1).

**Table 1:** Mean (SE) of BMI, WC and WHR in three phases of TLGS.

Age group (years)	Number	BMI(kg/m^2^)			WC (cm)			WHR		
		**Phase I**	**Phase II**	**Phase III**	**Phase I**	**Phase II**	**Phase III**	**Phase I**	**Phase II**	**Phase III**

***Men***										

20-29	266	24.6 (0.3)	26.0 (0.3)	27.1 (0.3)*	83.0 (0.8)	91.0 (0.8)	95.0 (0.7)*	0.86 (0.00)	0.91 (0.00)	0.94 (0.00)*

30-39	459	26.2 (0.2)	27.0 (0.2)	27.6 (0.2)*	88.3 (0.5)	94.4 (0.5)	96.2 (0.5)*	0.90 (0.00)	0.93 (0.00)	0.96 (0.00)*

40-49	369	26.6 (0.2)	27.2 (0.2)	27.4 (0.2)*	90.8 (0.5)	95.7 (0.5)	97.0 (0.5)*	0.93 (0.00)	0.95 (0.00)	0.97 (0.00)*

50-59	319	26.5 (0.2)	26.8 (0.2)	26.4 (0.2)*	92.1 (0.6)	96.2 (0.6)	97.4 (0.5)*	0.95 (0.00)	0.97 (0.00)	0.99 (0.00)*

60-69	328	26.5 (0.2)	26.7 (0.2)	26.5 (0.2)†	93.0 (0.6)	97.1 (0.6)	97.7 (0.6)*	0.95 (0.00)	0.98 (0.00)	1.00 (0.00)*

≥ 70	94	25.4 (0.4)	25.7 (0.4)	25.5 (0.4)	90.7 (1.0)	94.9 (1.0)	95.7 (0.9)*	0.95 (0.00)	0.98 (0.00)	0.99 (0.00)*

**Total**	**1835**	26.1 (0.1)	26.7 (0.1)	27.1(0.1)*	89.6 (0.3)	95.0 (0.2)	96.6 (0.2)*	0.92 (0.00)	0.95 (0.00)	0.97 (0.00)*

***Women***										

20-29	442	24.4 (0.2)	25.8 (0.2)	26.4 (0.2)*	78.0 (0.5)	81.5 (0.5)	81.4 (0.5)*	0.77 (0.00)	0.80 (0.00)	0.80 (0.00)*

30-39	629	27.2 (0.2)	28.5 (0.2)	28.7 (0.2)*	85.2 (0.4)	89.1 (0.4)	88.7 (0.5) *	0.81 (0.00)	0.84 (0.00)	0.84 (0.00)*

40-49	614	29.2 (0.2)	30.1 (0.2)	30.4 (0.2)*	91.5 (0.5)	95.0 (0.4)	95.0 (0.4)*	0.85 (0.00)	0.88 (0.00)	0.88 (0.00)*

50-59	509	29.6 (0.2)	30.2 (0.2)	30.5 (0.2)*	94.4 (0.5)	97.8 (0.5)	97.7 (0.5)*	0.88 (0.00)	0.91 (0.00)	0.91 (0.00)*

60-69	327	28.8 (0.2)	29.4 (0.2)	29.3 (0.2)*	95.0 (0.6)	98.0 (0.6)	97.2 (0.6)*	0.91 (0.00)	0.93 (0.00)	0.93 (0.00)*

≥ 70	46	27.0 (0.6)	27.4 (0.6)	27.4 (0.6)†	92.7 (1.7)	95.0 (1.6)	94.3 (1.8)	0.91 (0.01)	0.94 (0.01)	0.94 (0.01)*

**Total**	**2567**	27.9 (0.1)	28.9 (0.1)	29.1 (0.1)*	88.7 (0.2)	92.2 (0.2)	91.9 (0.3)*	0.84 (0.00)	0.87 (0.00)	0.87 (0.00)*

SE: Standard Error, BMI: Body Mass Index, WC: Waist Circumference, WHR: Waist to Hip Ratio, TLGS: Tehran Lipid and Glucose Study,

* p < 0.001; † p < 0.01 as compared to values in Phase I.

### Sex-specific prevalences of overweight, obesity and abdominal obesity

Overall prevalence of overweight, obesity and abdominal obesity in both sexes are showed in Table 2; while age adjusted prevalence of overweight, obesity and abdominal obesity in baseline were 55.4, 14.4 and 303% in men and 64.5, 22.9 and 68% in women, respectively. Percentages of overweight, obesity and abdominal obesity have risen significantly in both sexes through three phases mentioned, (p < 0.001) (Table 2). In phase III, The highest prevalence of overweight and abdominal obesity was found in the 40-49 and 60-69 year-old groups of both sexes, respectively. The maximum prevalence of obesity was seen in 30-39-year-old men and 50-59-year old women. Although a higher prevalence of abdominal obesity was seen in women (p < 0.001), a slight decrease was observed in phase III.

**Table 2:** The prevalences of Overweight, Obesity and Abdominal obesity in three phases of TLGS.

Age group (years)	Number	Overweight, %			Obesity, %			Abdominal obesity, %		
		**Phase I**	**Phase II**	**Phase III**	**Phase I**	**Phase II**	**Phase III**	**Phase I**	**Phase II**	**Phase III**

***Men***										

20-29	266	40.6	55.3	67.3*	10.5	17.6	22.9*	16.1	38.7	53.7*

30-39	459	59.9	69.0	74.3*	15.9	30.7	24.6*	32.6	54.6	61.4*

40-49	369	67.5	74.6	75.1*	15.2	50.3	22.77*	37.6	61.5	65.0*

50-59	319	66.8	69.0	70.8	20.7	47.9	19.7	45.7	62.3	67. 7*

60-69	328	66.8	66.5	66.5	16.8	41.8	16.5	48.4	66.4	69.2*

≥ 70	94	52.1	54.2	48.9	13.8	19.6	12.8	35.1	55.3	57.4*

**Total**	**1835**	**60.6**	**66.9**	**70.1**	**15.8**	**37.8**	21.1*	**36.5**	**57.2**	63.3*

***Women***										

20-29	442	41.8	55.7	59.0*	11.8	17.6	18.1*	43.8	54.9	55.2*

30-39	629	69.3	78.3	80.9*	23.2	30.7	33.7*	68.9	80.2	79.8*

40-49	614	82.7	88.3	89.6*	40.4	50.3	49.0*	86.3	92.1	92.5*

50-59	509	84.3	88.2	88.6*	44.4	47.9	52.0*	91.9	94.6	93.9†

60-69	327	81.6	83.1	82.9	38.2	41.8	40.9	92.3	95.7	95.4†

≥ 70	46	73.9	78.3	73.9	17.4	19.6	26.7	89.1	93.4	89.1

**Total**	**2567**	**72.4**	**79.4**	80.9*	**31.3**	**37.8**	38.6*	**76.7**	**83.8**	83.6*

TLGS: Tehran Lipid and Glucose Study, Overweight: BMI = 24.9-29.9, Obesity: BMI ≥ 30, Abdominal obesity: WC ≥ 94/80 cm for men/women respectively, * p < 0.001, † p < 0.01 as compared to values in Phase I

### Changes in BMI, WC and WHR in both sexes

**Figure 1:**
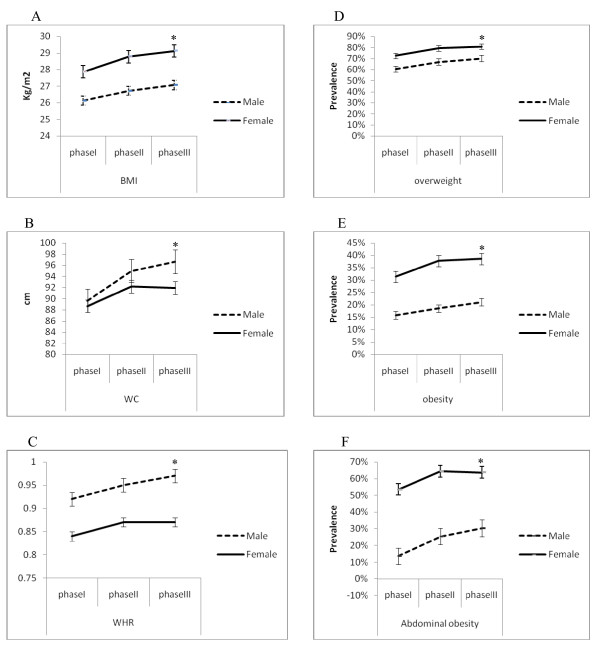
**Median 6.6-years changes in BMI(A), WC(B), WHR(C), and prevalences of overweight(D), obesity(E) and abdominal obesity(F)**. BMI: Body Mass Index, WC: Waist circumference, WHR: Waist to Hip Ratio, Overweight: BMI = 24.9-29.9, Obesity: BMI ≥ 30, Abdominal obesity: WC ≥ 94/80 cm for men/women respectively * p < 0.001 as compared to values in Phase I.

The trends of changes in BMI, WC and WHR from phase I to II showed increases in both sexes, significantly more so in women. Among men, this trend increased from phase II to III, while in women it was relatively stable (Figure 1A, B, C). Trends of overweight, obesity and abdominal obesity in women increased from phases I to III, the rate decreasing between phases II and III in women, but not so in men (Figure 1D, E, F).

**Figure 2:**
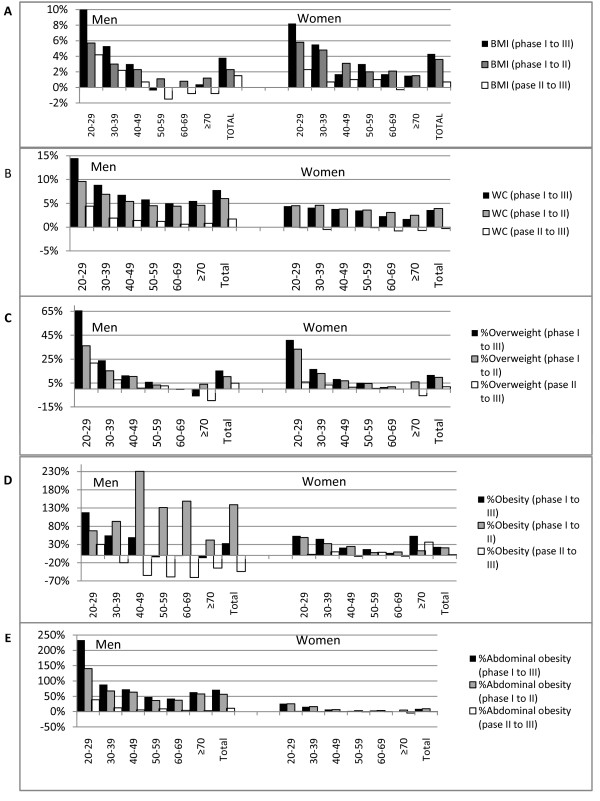
**Median 6.6-years, age-group specific changes in BMI(A), WC(B) and the prevalences of overweight(C), obesity(D) and abdominal obesity(E)**. BMI: Body Mass Index, WC: Waist circumference, Overweight: BMI = 24.9-29.9, Obesity: BMI ≥ 30, Abdominal obesity: WC ≥ 94/80 cm for men/women respectively.

Figure 2 shows relative changes in BMI, WC, overweight, obesity and abdominal obesity in the different age groups of men and women. Among both sexes, the highest rise in BMI was observed among 20-29 year-olds, with a smaller peak being observed among 30-39-years-olds. In men, changes in WC were substantially larger between phases I and II than between II and III, a trend not observed in women (Figure 2A, B). Men showed a greater change in prevalence of overweight, obesity and abdominal obesity, particularly in the youngest age group (20-29 years) (Figure 2C, D, E).

An association was observed between female gender and overweight, obesity and abdominal obesity in all 3 phases, but the strength of this association decreased between phase II and III (p < 0.001) (Table 3).

**Table 3:** The OR(95% CI) of overweight, obesity and abdominal obesity in women(n = 2567) compared to men(n = 1835) in three phases.

	Odds ratio (95% CI)		
	**Phase I**	**Phase II**	**Phase III**

**Overweight**:			

**Men**	**1.0**	**1.0**	**1.0**

**Women**	**1.7 **(1.5-1.9)	**1.9 **(1.7-2.2)	**1.8 **(1.6-2.0)

**Obesity**:			

**Men**	**1.0**	**1.0**	**1.0**

**Women**	**2.4 **(2.0-2.8)	**2.6 **(2.3-3.0)	**2.3 **(2-2.6)

**Abdominal Obesity**:			

**Men**	**1.0**	**1.0**	**1.0**

**Women**	**5.7(**5.0-6.5)	**3.9**(3.4-4.4)	**2.9**(2.5-3.4)

OR: odds ratio, CI: confidence interval, Overweight: BMI = 24.9-29.9, Obesity: BMI ≥ 30, Abdominal obesity: WC ≥ 94/80 cm for men/women respectively.

## Discussion

The results of this study show an increasing trend of obesity and abdominal obesity in the urban adult Tehranian population over 6.6 years. The prevalence of obesity increased 33 and 23% in men and women respectively; abdominal obesity during this period, showed increase of 71% in men and 9% in women. Thus, the increasing trend of abdominal obesity was dramatically higher in men than in women; also at the end of follow up the prevalences of abdominal obesity in men and women were 63 and 84%, respectively.

This high increase in the prevalence of obesity and abdominal obesity may be due to changes in lifestyle and shifts in nutritional patterns in Iran, [15] attributable to industrialization and substitution of high-fat, refined carbohydrate, and low-fiber diets [16]. The shift in the frameworks of occupations, transportation systems and the nature of activities related to occupations and leisure both in Iran [17] and worldwide, [18] has led to lower levels of physical activity, a main contributor to the increasing trend in obesity and abdominal obesity. In addition, we found that the distribution of prevalence of obesity did not change much, and a remarkable shift towards higher prevalence of abdominal obesity was observed. These findings are in agreement with literature available from USA [6] and Finland [19]. Explanations for the upward trend in abdominal obesity in comparison with fewer changes in obesity may be related to the changes in health behaviour over time. Several studies have shown lifestyle factors to be associated with body fat distribution [19], for example a decrease in physical activity level, increased smokers and changes in trans fat and fiber consumption [20].

Recent studies show the trend of obesity and abdominal obesity to be increasing, in both developed and developing countries [18,19,21]. This trend rose from 12 to 23% in USA over 15 years [22] and from 43 to 52% in Jordan over 10 years [23], as a developing country. Our findings indicate that the prevalence of Tehranian obese adults is increased from 25 to 31% during 6.6 years; the difference may be due to varying cultures of societies, the socio-economic status of populations and design and sample size of studies. Although the trend of abdominal obesity is seen to be increasing in all studies, describing and comparing the prevalence of abdominal obesity in different populations is somewhat problematic because different cut points have been use in different studies.

In agreement with other studies worldwide[1,24] and in Iran [7,9,25], our findings show that the prevalences of obesity and abdominal obesity in women are higher than men; gender differences in food intake, physical activity level and psycho-socio-economic status may be responsible for this difference. For example, results of a survey showed that Tehranian women consume more sweets and simple sugars [26]. In addition, lower physical activity levels among women, several pregnancies, lower socio-economic status, unemployment in many Tehranian women, a higher ratio of depression, and lower education level may be some of the reasons for the susceptibility of women to obesity as compared to men [17]. In contrast of our results, the prevalence of obesity in Kuwait [27] and abdominal obesity in Palestin among women was lower than men [28]; which can be due to differences in cultures of the Arab and Iranian populations.

In this study, we provide evidence that the trends of obesity and abdominal obesity are more alarming in men than in women, which can be due to the importance given by women to their health, because of their increasing educational levels and more income in recent years. It can also be a result of public educational programs being focused more on women as the target group because of their higher obesity levels compared to men. Moreover ignoring men in implemented public educational programs, causes the trend of obesity got worse among them compared to women during recent years. Our findings are consistent with recently published studies from Sweden [29,30], England [31] and Greece [32], showing the upward trends both in general and abdominal obesity among men (especially in the youngest group), compared to these levels remaining stable among women during recent years.

Regarding both the strengths and limitations of our study, the main strength of our study is first large population-based study with 6.6 years follow up of a same population in Iran, and the Middle East Region. The main limitation of our analyses, a considerable fraction of subjects (about fifty percent) was lost during the follow-up period due to immigration. Considering that those who lost to follow up had lower BMI and WC compared to those who completed study, we may have overestimated the reported trend in overweight, obesity and abdominal obesity in this population.

## Conclusion

In conclusion, an alarming increase was shown in the prevalences of overweight, obesity and abdominal obesity among in both sexes of Tehranian adults, the highest trend being observed in the 20-29 year age groups, especially in men. There is the urgent need to target younger ages for prevention and implementation of public educational programs to curtail this rising trend in obesity and abdominal obesity especially in young men.

## Competing interests

The authors declare that they have no competing interests.

## Authors' contributions

FH participated in the conception and design of the study, coordination and its final approval. MB participated in its design, performed the statistical analysis and drafted the manuscript. PSE helped in statistical analyses of the study. PM and FA revised the manuscript for important intellectual content. All authors read and approved the final manuscript.

## Pre-publication history

The pre-publication history for this paper can be accessed here:

http://www.biomedcentral.com/1471-2458/9/426/prepub
